# Focal nodular hyperplasia‐like lesion in a girl with obesity and fatty liver

**DOI:** 10.1111/ped.15392

**Published:** 2022-12-23

**Authors:** Mie Mochizuki, Yasuhiro Nakayama, Kazumasa Sato, Takeshi Inukai

**Affiliations:** ^1^ Department of Pediatrics Kyonan Medical Center Fujikawa Hospital Yamanashi Japan; ^2^ Department of Pediatrics University of Yamanashi Yamanashi Japan; ^3^ Department of Pediatrics National Hospital Organization, Kofu Hospital Yamanashi Japan; ^4^ Department of Internal Medicine Kyonan Medical Center Fujikawa Hospital Yamanashi Japan

**Keywords:** children, fatty liver, focal nodular hyperplasia, hyperinsulinemia, obesity

Focal nodular hyperplasia (FNH) is a noncirrhotic tumor that is the second most benign tumor, accounting for 2% of all pediatric liver tumors.^1^ On diagnostic imaging, FNH appears as vascular liver masses that must be differentiated from hepatocellular carcinoma, hepatocellular adenoma, cavernous hemangioma, and polycystic metastatic liver cancer.

A 13‐year‐old Japanese girl developed a nodular hepatic lesion that was undetectable on ultrasonography 12 months ago. She was initially treated as an outpatient for obesity at 8 years of age. At 10 years old, she had obesity (obesity index 53.3%) and fatty liver confirmed by ultrasonography. Abdominal ultrasonography had been performed annually since then.

Her medical history included successful treatment for Kawasaki disease with high‐dose immunoglobulin therapy. She had also occasionally received leukotriene antagonist for bronchial asthma as a 1‐year‐old and had received midodrine hydrochloride because of orthostatic dysregulation since she was 10 years old. No bleeding tendency was observed. Her parents had bronchial asthma and her brother had childhood obesity, but there was no family history of liver disease.

On physical examination, her height was 158.8 cm (+0.8 SD) and weight was 81.6 kg (+2.9 SD). Her obesity index and body mass index were 61.0% and 32.4, respectively, indicating obesity. Her abdominal circumference was 113 cm and blood pressure was 112/70 mmHg. She had acanthosis nigerians on her neck and axilla and striae distensae on her abdomen, buttocks, and thighs.

Blood examination indicated moderately increased aminotransferase levels due to fatty liver (AST 48 IU/L, ALT 102 IU/L, LDH 254 IU/L, total bilirubin 0.45 mg/dl) and hyperinsulinemia with a normal HbA1c (5.2%), normal fasting plasma glucose (96 mg/dl), and elevated immunoreactive insulin level (45.2 mIU/ml). Her FIB‐4 index based on her age, AST and ALT levels, and platelet cell count (452,000/μl) was 0.14, which was not indicative of liver fibrosis. Hepatitis B and C markers (Hbs‐Ag, Hbs‐Ab, HCV‐Ab) and tumor markers (CEA, AFP, CA 19–9, S‐PIVKA‐II, DUPAN‐2) were within normal ranges.

Ultrasonography revealed a hypoechoic nodule with clear boundaries in S7 and uniform internal and enhanced posterior echoes (Figure 1a‐1). Perflubutane‐enhanced ultrasonography showed that the mass extended from the center to the periphery in the arterial phase with prolonged contrast time (Figure 1a‐2,3), and the same contrast as the background in the posterior vascular phase (Figure 1a‐4). Computed tomography revealed fatty liver and a solitary 28‐mm‐diameter homogeneous nodule in S7 (Figure 1b‐1). Dynamic computed tomography showed an enhanced mass in the early arterial phase (Figure 1b‐2); the contrast of the mass was weakened in the late phase but remained more marked than that of the surrounding tissue (Figure 1b‐3). Magnetic resonance imaging (MRI) revealed a nodule with high signal intensity. Gadoxetate disodium‐methoxybenzyl‐diethylenetriamine pentaacetic acid‐enhanced MRI showed a solitary mass with poor enhancement at the center (Figure 1c‐2), a slightly enhanced signal on T2‐weighted imaging (Figure 1c‐3), and a central scar‐like appearance in the hepato‐biliary phase (Figure 1c‐4), which are typical characteristics of FNH.^2^, ^3^, ^4^ Fluorodeoxyglucose‐positron emission tomography showed no abnormal fluorodeoxyglucose accumulation (data not shown). Although no pathological examination was performed, a clinical diagnosis of FNH‐like lesion was made based on imaging. Ultrasonography performed 7 and 13 months later and MRI performed 15 months later showed that the hepatic nodule size was unchanged without specific therapy.

**FIGURE 1:**
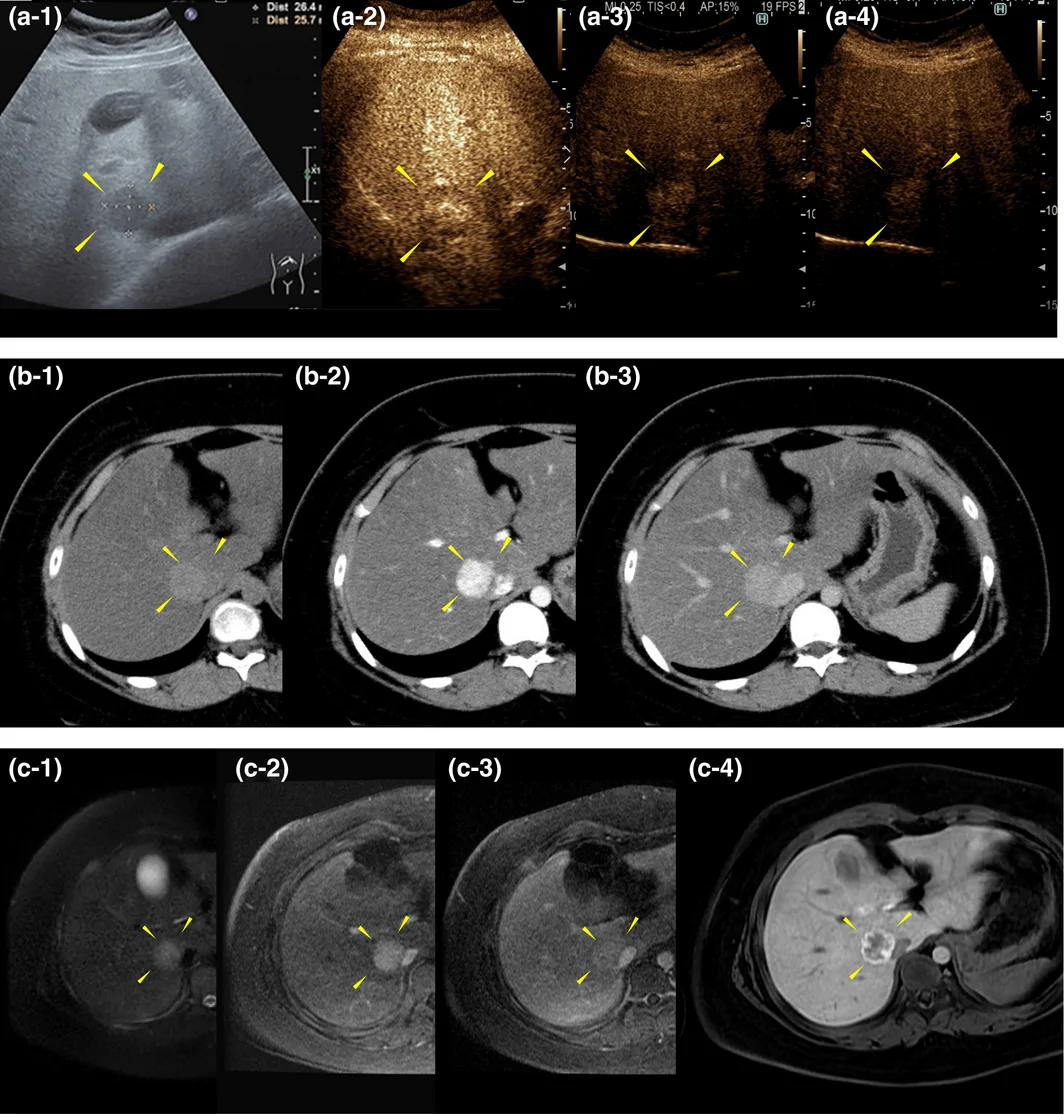
Liver imaging findings. (a) Ultrasonographic images. a‐1, B‐mode; a‐2, arterial phase; a‐3, portal vein phase; a‐4, post vascular phase in harmonic tissue by perflubutane contrast‐enhanced imaging. (b) Enhanced computed tomography images. b‐1, nonenhanced; b‐2, arterial phase; b‐3, late phase. (c) Gadoxetate disodium‐methoxybenzyl‐diethylenetriamine pentaacetic acid contrast‐enhanced magnetic resonance images. c‐1, nonenhanced T2‐weighted; c‐2, arterial phase; c‐3, portal vein phase; c‐4, hepato‐biliary phase. The yellow marks indicate the FNH‐like lesion.

Focal nodular hyperplasia is most common in women aged 30–50 years with no liver disease, and often accompanies abnormal vascular morphology. Pediatric patients with FNH often have a history of anticancer drugs and radiation therapy.^1^, ^5^

A definitive diagnosis of FNH is based on (1) absence of liver damage, (2) spoke‐wheel appearance of the vascular structure and a central scar on imaging, and (3) pathological findings of central scar‐like fibrous tissue, increased cell density, and hyperplasia.^2^, ^3^, ^4^ Malignant change is rare, and the prognosis is good.^2^ Similar nodules in patients with some liver disease or atypical imaging or pathological findings are diagnosed as FNH‐like lesions.^4^

The Central Medical Journal has reported 73 cases of FNH and two of FNH‐like lesions in children <18 years since 1985, but no FNH or FNH‐like lesions in children with fatty liver. Epidemiological data also suggested that being overweight or obese was associated with liver cancer.^6^ Furthermore, the risk of progression to cirrhosis and hepatocellular carcinoma has been reported in nonalcoholic hepatitis.^7^ This case was FNH with nonalcoholic fatty liver, and will be followed up with serial examinations including clinical examinations every 6 months using the protocol for the follow‐up of FNH.^5^

We encountered a girl with an FNH‐like lesion and fatty liver. Follow‐up is necessary because the lesion appeared within 1 year and she has a fatty liver and hyperinsulinemia.

## AUTHOR CONTRIBUTIONS

M.M. was responsible for managing the patient, review of the clinical chart, perusing the potentially relevant literature, and drafting the initial manuscript, and conceiving the case report. M.M. and Y.N. were responsible for planning and implementing diagnostic imaging. Y.N., K.S., and T.I. supervised the draft manuscript. All authors read and approved the final manuscript.

## CONFLICT OF INTEREST

The authors declare no conflict of interest.

## INFORMED CONSENT

Written informed consent was obtained from the patient's guardian for the publication of this manuscript.
